# Modern Catalytic Materials for the Oxygen Evolution Reaction

**DOI:** 10.3390/molecules30081656

**Published:** 2025-04-08

**Authors:** Michał Trębala, Agata Łamacz

**Affiliations:** Department of Engineering and Technology of Chemical Processes, Wroclaw University of Science and Technology, Gdanska 7/9, 50-344 Wroclaw, Poland

**Keywords:** metal–organic frameworks, noble metals, oxygen evolution reaction, transition oxide

## Abstract

The oxygen evolution reaction (OER) has, in recent years, attracted great interest from scientists because of its prime role in a number of renewable energy technologies. It is one of the reactions that occurs during hydrogen production through water splitting, is used in rechargeable metal–air batteries, and plays a fundamental role in regenerative fuel cells. Therefore, there is an emerging need to develop new, active, stable, and cost-effective materials for OER. This review presents the latest research on various groups of materials, showing their potential to be used as OER electrocatalysts, as well as their shortcomings. Particular attention has been paid to metal–organic frameworks (MOFs) and their derivatives, as those materials offer coordinatively unsaturated sites, high density of transition metals, adjustable pore size, developed surface area, and the possibility to be modified and combined with other materials.

## 1. Introduction

One of the biggest challenges scientists face today is the creation of new environmentally safe energy sources. This challenge arises due to the rapidly increasing demand for energy, which is expected to double in the near future [1]. As energy is produced mainly from fossil fuels, the need to develop more environmentally friendly technologies and devices—such as water splitting [2], metal–air batteries [3] and fuel cells [4]—has become an important issue for both academia and modern industry. Water splitting is considered a promising method for producing hydrogen and oxygen, including the hydrogen evolution reaction (HER) and the oxygen evolution reaction (OER) [5]. The main advantage of this method of producing hydrogen is that it does not emit carbon dioxide, making it a “green” technology, and the resulting hydrogen can be classified as “green” [6]. Green H_2_ is set to become a significant force in the future of energy, enabling comprehensive decarbonization in various sectors and enhancing energy security. It is a vital element of worldwide sustainability initiatives. To fully realize its potential and integrate it into primary energy infrastructures, strategic investments and coordinated policy strategies will be crucial.

However, both reactions (HER and OER) exhibit slow kinetics and high activation energy barriers, necessitating the use of catalysts to enhance efficiency and feasibility. The HER, which involves the reduction of protons to form molecular hydrogen, requires an efficient catalyst to facilitate electron transfer and lower the overpotential. In acidic media, the reaction follows either the Volmer–Heyrovsky or Volmer–Tafel mechanism [7], where the formation of adsorbed hydrogen intermediates (*H) is a key rate-limiting step. In alkaline media, HER is further hindered by the additional energy required for water dissociation. Platinum-based materials remain the most effective catalysts because of their optimal hydrogen binding energy. The OER, a four-electron oxidation process, is even more kinetically sluggish due to the complex formation of oxygen-containing intermediates (*OH, *O, *OOH). Without a catalyst, the OER exhibits a high activation energy, making the reaction inefficient. Transition metal oxides (e.g., IrO_2_, RuO_2_ in acidic medium and Ni-Fe oxides in alkaline medium) significantly enhance the reaction rate by stabilizing intermediates and reducing overpotential. Overall, catalysts are essential for overcoming kinetic limitations, reducing energy losses, and improving the long-term stability of electrochemical water splitting systems, thereby enabling practical hydrogen production for energy applications [8,9].

In reversible fuel cells and metal–air batteries, the OER is accompanied by the oxygen reduction reaction (ORR) [2,3,4]. Therefore, the development of efficient, robust, and inexpensive OER electrocatalysts is of particular importance for the progress of advanced and clean technologies for energy generation and storage.

The OER is a slow, pH-dependent reaction with multiproton/electron-coupled steps [10] At 25° C and 1 atm. The equilibrium half-cell potentials (E^0^_a_) for the OER are as follows:

in alkaline medium: 4OH^−^ 2H_2_O (l) + O_2_(g) + 4e^−^ E^0^_a_ = 0.404 V (1)

in acidic medium: 2H_2_O_(l)_ ⟷ 4H^+^ + O_2(g)_ + 4e^−^ E^0^_a_ = 1.23 V (2)

To overcome the kinetics of electron–proton transfer, a large overpotential is required [11]. For conducting the OER at pH = 0 one needs to create the potential difference of 1.23 V vs. the normal hydrogen electrode external; therefore, an external current is needed. The pH influences the applied potential, and, according to the Nernst equation, the shift in the reaction potential per unit of pH is ca. 59 mV. By using the reversible hydrogen electrode (RHE) as a reference electrode, the working voltage can be kept around 1.23 V during the OER.

**Figure 1:**
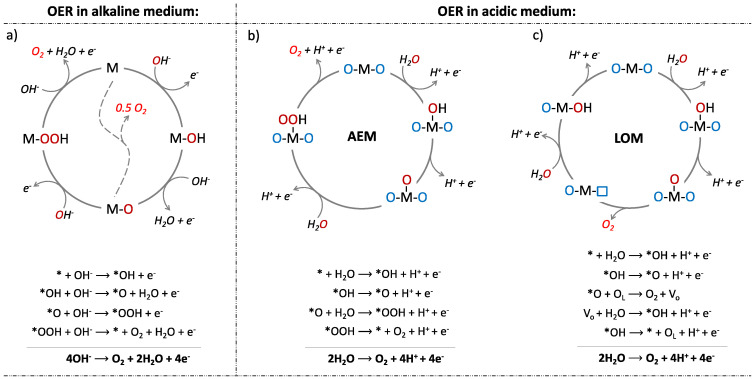
The mechanisms of OER (water oxidation reaction) under alkaline (**a**) or acidic conditions (**b**,**c**).

The mechanisms of OER under alkaline or acidic conditions differ significantly (Figure 1). Alkaline-condition OER catalysts offer a good combination of cost efficiency, material stability, and tunable reactivity, making them more promising for next-generation energy technologies. Advances in catalyst design and mechanistic understanding will further bridge the gap toward industrial-scale implementation [12].

In alkaline medium (Figure 1a), an electrolyte hydroxyl anion (OH^−^) is first adsorbed at the active site (M), and, in a one-electron oxidation, produces oxo intermediates (M-OH), whose reaction with another OH^−^ gives a molecule of M-O and H_2_O. The direct reaction of two M-O intermediates leads to the regeneration of M (dashed line). Alternatively, the reaction of M-O with OH^−^ produces peroxide intermediates (M-OOH), which, after reacting with another OH^−^, transform into M, O_2_, and H_2_O.

The mechanism of the OER in acidic media is not fully understood, but two primary mechanisms have been proposed. Both mechanisms depend on the sources of oxygen evolution. The first is called the “adsorbate evolution mechanism” (AEM), which uses oxygen from H_2_O in the electrolyte, while the second is known as the “lattice oxygen participation mechanism” (LOM) (Figure 1b,c). In both mechanisms, the first step is the nucleophilic attack of water on the metal active site (M), followed by deprotonation to form *OH and *O intermediates (M-OH and M-O, respectively). According to a DFT study by Rossmeisl et al. [13,14] in AEM, the *O intermediate (M-O) is then attacked by another H_2_O molecule to form the *OOH intermediate (M-OOH), which undergoes deprotonation to give oxygen and renovate the active site M. In LOM, the *O intermediate (M-O) couples with adjacent lattice oxygen to form gaseous O_2_. The oxygen vacancy (V_0_), which is also formed in this step, acts as an active site for a water nucleophilic attack, leading to the *OH intermediate (M-OH). After deprotonation, the metal active site is regenerated [15].

To evaluate the kinetics and mechanism of the electrocatalytic reaction and to compare different catalysts, the overpotential (*η*) and Tafel slope are used. The Butler–Volmer equation is one of the most fundamental relationships in electrochemical kinetics.

(3) $$i=i_{0}(e^{\frac{\alpha_{a}zF\eta }{RT}}-e^{e^{\frac{\alpha_{c}zF\eta }{RT}}})$$

In which are the following:

*i*— the current density (A·m^−2^),
*i*_0_— the exchange current density (defined as the reduction reaction rate and the oxidation reaction rate at equilibrium potential) (A·m^−2^),
α*_a_*— the anodic charge transfer coefficient,
α*_c_*— the cathodic charge transfer coefficient,
*z*— the number of electrons involved in electrode reaction,
*F*— the Faraday constant (96,485 C·mol^−1^),
*η*— activation overpotential defined as the difference between the potential necessary to practically run the reaction (E) and the theoretically found equilibrium potential of the reaction (E_eq_) (V),
*R*— the universal gas constant (8.31 J·mol^−1^·K^−1^),
*T*— temperature (K).

The overpotential is one of the key factors for assessing the performance of an OER catalyst. For the high overpotential at the anode, Equation (3) simplifies to the Tafel equation (Equation (4)), which, after taking the logarithm (Equation (5)), allows one to determine the Tafel slope (Equation (6)), i.e., the coefficient b in Equation (5).

(4) $$i\approx i_{0}e^{\alpha_{a}F\eta }$$

(5) $$\log\left(i\right)\approx \log\left(i_{0}\right)+\frac{\eta }{b}$$

(6) $$b=\frac{2.303RT}{\alpha F}$$

The Tafel slope represents the amount of change required for an overpotential when the current changes ten times. It shows how fast the current increases with the applied overpotential. It also helps to find the rate-determining step (RDS) and to determine the mechanism for the reaction. In the case of the OER, the RDS can be both single- and multi-electron. The transfer coefficient *α* for a one-electron reaction is the same as the symmetry coefficient β and is described by Equation (7).

(7) $$\beta =\alpha =\frac{1}{2}+\frac{\eta }{\lambda }$$

The value of the energy reorganization coefficient (*λ*) is much greater than the overpotential (*η*), so the transfer coefficient for a single electron reaction is approximately 0.5. If this assumption is valid, one can calculate the Tafel slope for a single-electron reaction based on Equation (7) and yielding a value of 120 mV dec^−1^. In most cases, the situation is more complicated and the reaction takes place in many stages. And the transfer coefficient *α_a_* is described by the equation, according to Bockris [16].

(8) $$\alpha_{a}=\frac{\eta_{b}}{\nu }+\eta_{r}\beta$$

where $\eta_{b}$ is the number of electrons that are transferred back to the electrode before RDS, ν is the number of rate-determining steps that have occurred in the overall reaction, $\eta_{r}$ is the number of electrons that are involved in RDS, and *β* is the symmetry factor. In the case of four-electron reactions, such as the OER, the rate-determining step is the third electron transfer step, where $\eta_{b}$ and ν are equal to 2 and 1 ($\eta_{r}$ and *β* are 0), respectively. This yields a transfer coefficient of 2 and theoretical value Tafel slope of 30 mV·dec^−1^ [17]

A catalyst that exhibits high activity in the OER reaction must be characterized by a low value for the Tafel slope. The smaller the Tafel slope, the smaller the voltage increase and the lower the energy consumption. In the last 10 years, research on the oxygen evolution reaction has been extensive, with particular emphasis on finding efficient and cost-effective catalysts for this reaction. An interesting alternative to noble metal catalysts (the best catalysts for the OER reaction) is the use of catalysts made from metal–organic framework (MOF) structures and their derivatives.

**Figure 2:**
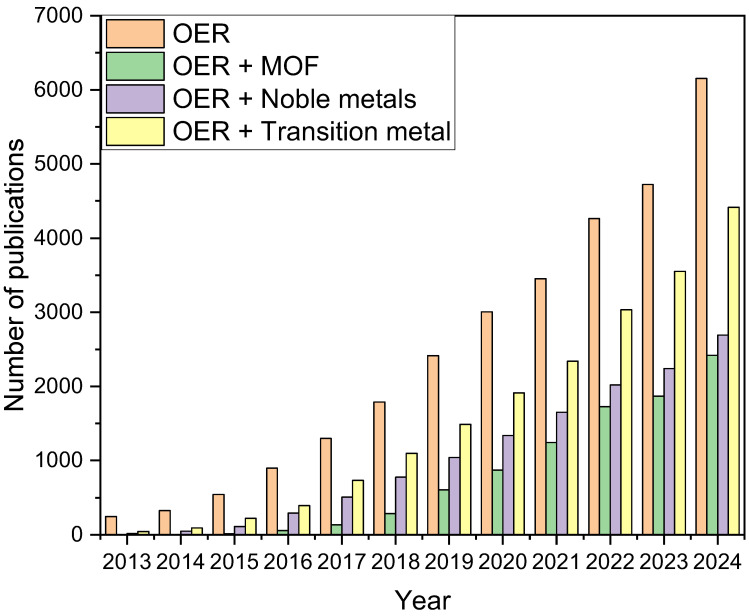
The number of papers published in recent years on OER and the use of MOF-based catalysts in this reaction.

Figure 2 shows the number of publications in recent years on OER and the use of MOF-based catalysts in this reaction. 

## 2. Noble Metals

In 1979, Beni et al. [18] showed that a catalyst based on a sputtered layer of iridium oxide had much higher activity in the reaction OER than catalysts based on other metals Since then, only ruthenium-based catalysts [19] have shown higher activity, but exhibit less stability than the iridium catalyst. Currently, catalysts based on iridium and ruthenium are the best for the OER due to the balance between stability and activity in acidic media [20]. Both elements are one of the rarest metals on Earth, with roughly 9 tons of Ir [21] and 30 tons of Ru [22].

Currently, the highest activity has been achieved by a catalyst based on iridium synthesized by Fan et al. [23] and made from a new metastable phase of 3R iridium oxide. The 3R-IrO_2_ achieves an ultralow overpotential of 188 mV at a current density of 10 mA·cm^−2^ and a Tafel slope of 52 mV·dec^−1^. Whereas the best performing ruthenium catalyst is Mn-RuO_2_ [24] that produces an overpotential of 158 mV at a current density of 10 mA·cm^−2^ and a Tafel slope of 43 mV·dec^−1^.

Due to the low availability of iridium and ruthenium, research on the use of these metals in the OER focuses on reducing their quantity in a catalyst while increasing their efficiency. One approach is to synthesize compounds with diverse morphologies and large specific surface areas (e.g., nanorods, nanosheets, nanofilms, nanoparticles of various sizes, mesoporous structures, etc.). For example, Escudero-Escribano and colleagues [25] synthesized iridium nanoparticles (IrNPs) with a diameter of 2.5 nm in ethylene glycol and used it as a catalyst for OER in acidic media. These synthesized nanoparticles showed activity comparable to commercial catalysts based on IrNPs [25]. Although catalysts based on pure iridium nanoparticles exhibit high activity in the OER (very low overpotential), their stability remains a concern. As a result, research is being conducted to improve their stability [26]. For example, ultrasmall IrNPs supported on nitrogen-doped graphene were investigated as a catalyst for OER [27]. This catalyst showed high activity in the OER, and DFT calculations indicated that N-doped graphene can stabilize IrNPs and promote the electrochemical reactions occurring on them. Another approach to better utilizing expensive noble metals in catalysis is their atomic dispersion on a support. Li et al. [28] presented results for highly dispersed amorphous iridium nanoclusters on a carbon substrate. The catalyst obtained in this way was much cheaper than commercial catalysts based on iridium, yet it exhibited similar activity (low overpotentials of 290 mV and Tafel slopes of 55 mV·dec^−1^). To further reduce the use of iridium and ruthenium while obtaining a stable and active catalyst for the OER reaction, a high-entropy ruthenium-iridium-based oxide with abundant grain boundaries (GB) has been developed. An example of such a catalyst is M-RuIrFeCoNiO_2_, presented by Hu et al. [29]. Their findings show that the intentional introduction of external metal elements and GB would effectively alter the electronic structure and OER pathway of RuO_2_, resulting in enhanced activity and stability. For ruthenium-based catalysts, recent research is focusing on improving their stability. Jin et al. [30] obtained a Re_0.06_Ru_0.94_O_2_ catalyst using dynamic Re dopants that exhibits significantly increased activity and stability for acidic OER [30]. A comparison of the activity of noble metal-based catalytic materials for the OER reaction is presented in Table 1.

## 3. Transition-Metal Catalyst

Transition metal catalysts are composed of various earth-abundant transition elements, such as Fe, Co, Ni, Mn, Cu, Zn, and Mo. As most metal oxides are alkaline oxides, they tend to lose activity when exposed to high potential in neutral and acidic environments, which limits their application [40]. Nevertheless, these compounds remain attractive due to their low cost. The OER catalysts containing transition metals are divided into three types based on single metal oxides, doped single metal oxides, and metal oxides with specific structures (e.g., ABO_3_, AB_2_O_4_, A_2_B_2_O_6_). For single metal oxides, Trasatti [41] compiled data to determine the empirical activity trend in the reaction of oxygen evolution, as follows: RuO_2_ > IrO_2_ > MnO_2_ > NiO > Co_3_O_4_ > Fe_3_O_4_, corresponding to the enthalpy of the reaction MO_x_ + 1/2O_2_ ⇌ MO_x_ + 1, which is believed to be related to the strength of the M-O bond. DFT calculations by Calle-Vallejo et al. [42] showed a similar trend in the activity of transition metal oxides in this reaction. However, the calculations of NiO, CoO, FeO, and MnO by Man et al. [14] revealed a slightly different activity trend: Co_3_O_4_ > NiO > Mn_x_Oy. Co_3_O_4_ benefits from its promising activity, low cost, simple preparation, and high stability. Monteverde Videla et al. [43] demonstrated that different surface morphologies of cobalt spinel Co_3_O_4_, obtained via different synthesis methods (precipitation, solution combustion synthesis, and hard template method), result in catalysts with varying electrocatalytic activities in the OER. The best catalyst was synthesized using the hard template method, which provided the largest specific surface area of 48 m^2^·g^−^^1^. For this catalyst, the overpotential calculated at 10 mA·cm^−2^ was 440 mV, and the Tafel slope was 65 mV·dec^−1^. In another study, Shi et al. [44] showed that BO_x_-decorated Co_3_O_4_ nanowires were effective OER electrocatalysts, requiring only 328 mV to drive a current density of 10 mA cm^−2^ while exhibiting excellent structural stability. The research by [44] Li et al. [45] highlighted the effect of tellurium (Te) doping in cobalt spinel. The benefits of Te doping for electrocatalytic properties were attributed to its small particle size, which exposes more active sites and creates rich oxygen vacancies. These oxygen vacancies help regulate electron distribution, expedite charge transfer, and improve electronic conductivity, as evidenced by EIS measurements [45]. Masa et al. [46] investigated CoO_x_-based catalysts supported on a nitrogen-doped carbon matrix (NC) and demonstrated that Co_3_O_4_/NC catalysts exhibit better activity than regular CoO catalysts. They also showed much lower overpotential compared with state-of-the-art catalysts such as RuO_2_, IrO_2_, and Pt/C. Additionally, Stelmachowski et al. [47] studied cobalt oxides on a carbon substrate and found that nitrogen-doped mesoporous carbons are promising supports for cobalt-based OER catalysts. The amount of cobalt deposited, along with the presence of nitrogen and oxygen in the carbon structure, were found to be crucial factors in enhancing catalytic performance.

Another catalyst based on transition metal spinels was studied by Xie et al. [48]. This was an iron-cobalt oxide spinel modified with borate. This work reported a synthesis strategy to achieve synergistic enhancement of the catalytically active species, and the improved activities made it a highly effective and stable OER catalyst. Kim et al. [49] tested four types of spinel structural materials (Mn_3_O_4_, Fe_3_O_4_, MnFe_2_O_4_, and Mn_2_FeO_4_) in the OER reaction. The MnFe_2_O_4_ electrode in a 1.0 M KOH alkaline electrolyte demonstrated the best electrocatalytic activity, with an OER overpotential of 310 mV.

Studies have shown that one way to improve the catalytic activity of transition metal oxides in the OER reaction is by applying a magnetic field. This is because the ferromagnetic catalyst enhances oxygen production in the triplet state (the most stable form), which in turn leads to a reduction in overpotential [50]. An example of this is the study by Li et al. [51], who demonstrated that the overpotential of Co_3_O_4_/NF was reduced (from 308 mV for Co_3_O_4_ spheres in nickel form) as the magnetic field strength increased, reaching a minimum value of 252 mV at 125 mT.

Cobalt sulfur nanosheets produced by electrodeposition for OER were studied by Nan et al. [52]. These studies revealed a unique nanosheet-based flower structure, which provides high electrocatalytic efficiency. This material demonstrates the highest activity and improved stability, requiring a low overpotential of 310 mV to achieve a current density of 10 mA·cm^−2^. Furthermore, some catalysts with special morphologies have also been effectively synthesized, such as the CuCo_2_S_4_ nanosheets by Chauhan et al. [53] and the CoFeS_x_ nanovesicles by the Wang group [54].

The first report on the use of the pure metal phosphide Ni_2_P as a highly active catalyst for OER was published in 2015 by Hu’s group [55]. Electrochemical measurements showed that the Ni_2_P nanowire material is an excellent electrocatalyst for water oxidation under alkaline conditions. The synergistic effect of Ni and Co in phosphates was investigated and characterized in detail by Barwe et al. [56]. In 2021, Xu et al. [57] utilized Fe-Co-P multi-heterostructure catalysts on a nickel foam substrate. Impressively, this catalyst exhibited high OER activity, with an overpotential of 227 mV and a Tafel slope of 55 mV·dec^−1^.

A summary of the comparison of catalytic properties in the range of materials based on transition metals is provided in Table 2.

## 4. Metal–Organic Framework Catalyst

**Figure 3:**
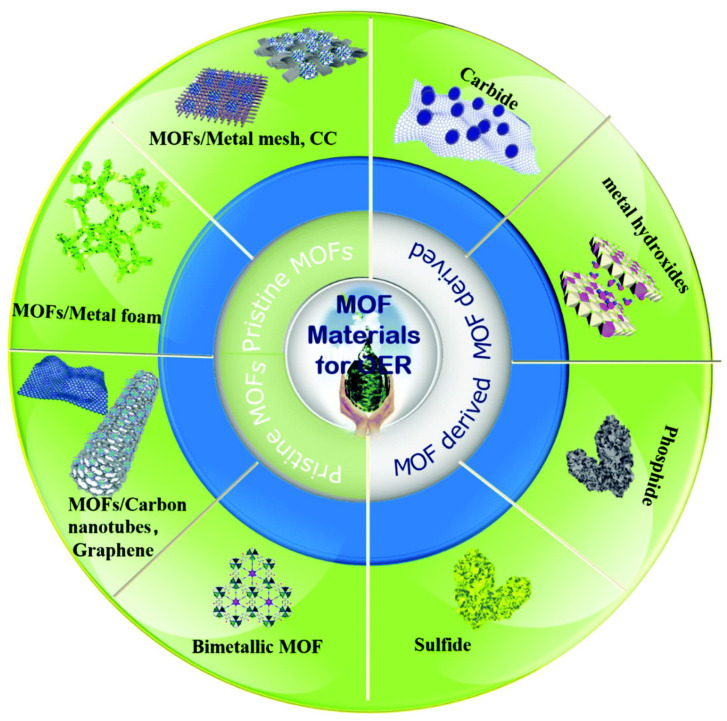
An overview of MOF-based materials for the oxygen evolution reaction. Reprinted from [58] with permission from RSC.

Metal–organic frameworks (MOFs) are a class of porous materials with periodic structures created by the self-assembly of transition metals (Fe, Co, Ni, Mn, Cu, Zn, and Mo) and organic ligands. They offer benefits such as high porosity, large specific surface area, flexible pore size, and diverse structures. The catalytic activity of MOFs is closely related to the role of metal atoms in the structure. The biggest challenges when using these structures for OER are their low conductivity and stability. As a result, extensive research has been undertaken to improve the conductivity of MOFs, including by combining them with highly conductive supports (e.g., graphene, polyaniline). Another method is the use of MOFs as precursors and templates for creating catalytic materials for OER [58]. Figure 3 provides an overview of the MOF-based materials used in OER.

### 4.1. Monometallic MOFs

**Figure 4:**
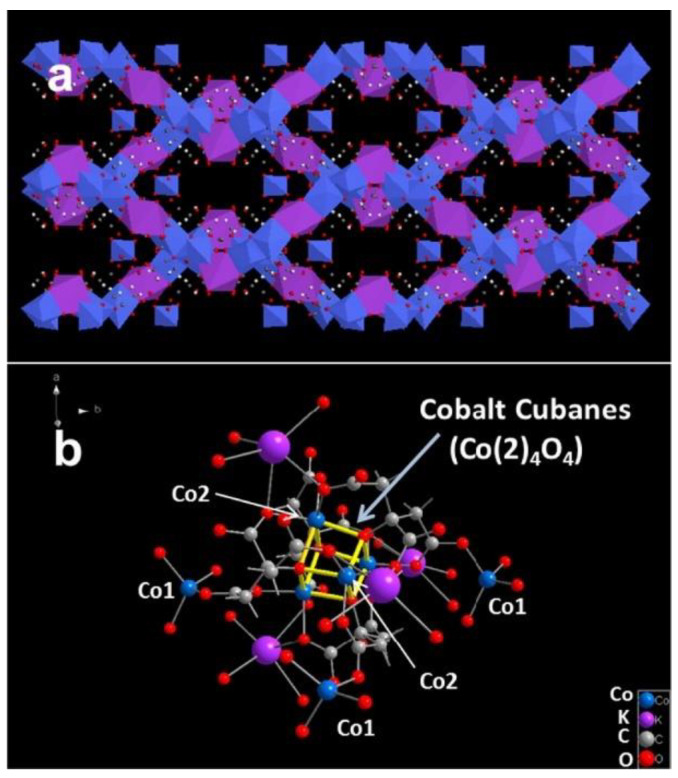
(**a**) Crystal structure of the UTSA-16 and (**b**) representative structures of Co_4_O_4_ cubane in the UTSA-16. Reprinted from [59] with permission from ACS.

The main problem with using monometallic MOF structures as a catalyst in the OER is their poor electrical conductivity. However, research is being conducted on MOFs containing transition metals. For example, Jiang et al. [59] synthesized the bioinspired cobalt−citrate metal−organic framework (UTSA-16 structure shown in Figure 4) and demonstrated its good performance in OER (current density of 10 mA·cm^−2^ at an overpotential of 408 mV and Tafel slope of 77 mV·dec^−1^).

**Figure 5:**
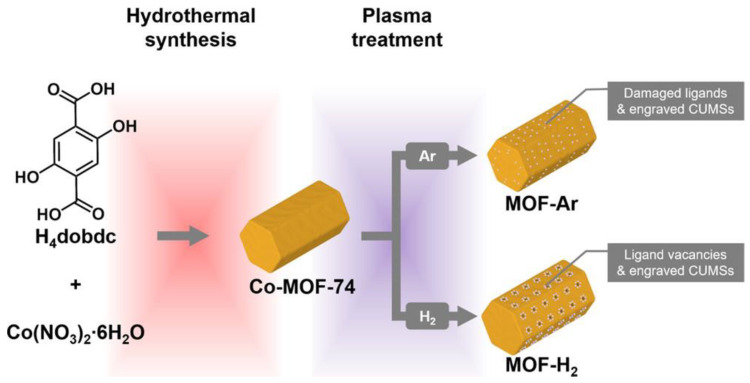
Schematic diagram of the preparation of catalysts by plasma. Reprinted from [60] with permission from ACS.

In addition, the researchers identified a synergistic effect associated with the presence of an open porous structure, high-valent cobalt, and the Co_4_O_4_ cubane in UTSA-16. Monometallic MOFs with cobalt metal centers were also studied by Zhu et al. [60]. Their research focused on the use of microwave-induced plasma to improve the electrocatalytic properties of monometallic Co-MOF-74 in the OER reaction. After optimizing the conditions of the argon and hydrogen plasma (the schematic diagram of catalyst preparation is shown in Figure 5), the resulting MOF structures exhibited improved electrocatalytic properties. The MOF treated with hydrogen plasma showed better OER activity in a basic electrolyte, with a low overpotential of 337 mV at 15 mA·cm^−2^.

Research on Fe-MOF nanosheets, synthesized via a low-temperature hydrothermal reaction and arrayed on NF, led by Zhang [61], showed excellent OER performance, requiring overpotentials of 240 and 270 mV to drive current densities of 50 and 100 mA·cm^−2^, respectively [61].

The work of Dymerska et al. [62] demonstrated that low-temperature calcination of ZIF-67 improves its electrocatalytic activity in the OER. The overpotential of ZIF-67 calcined at 200 °C is 317.8 mV, with a Tafel slope of 105.1 mV·dec^−1^.

The boron imidazolate framework (BIF) has also been studied as a catalyst for the OER. The advantage of these materials is their high chemical stability and the potential for a large number of electrocatalytically active centers (bound to both the metal center and the ligand). Zhang et al. [63] studied BIF-90 and demonstrated its OER activity, with an onset potential of 1.69 V and a current density of 10 mA·cm^−2^ at an overpotential of 460 mV.

A comparison of the electrocatalytic properties monometallic MOF structures in OER reactions is shown in Table 3.

### 4.2. Bimetallic MOF

The unsatisfactory performance of catalysts based on single-metal MOFs has sparked interest in bimetallic systems. The addition of a second transition metal to the MOF-based catalyst results in a strong synergistic effect. Bimetallic MOFs not only have improved electrical conductivity but also generate more active sites and increase stability. The greater number of active sites is due to the fact that different metals occupy different positions in the structure, creating distinct defects (e.g., the active sites of Co-doped Ni are not in the framework). Zhou et al. [64] showed DFT calculations and experimental tests for a series of novel isostructural transition-metal MOFs, [NH_2_(CH_3_)_2_][M_3_(μ_3_-OH)(H_2_O)_3_(BHB)] (M_3_ = Co_3_, Co_2_Ni, CoNi_2_, Ni_3_), for use as an OER catalyst. CoNi_2_ was the best combination among the four, with a calculated overpotential of 420 mV. Tang’s group [65] reported Ni-Co bimetallic organic framework nanosheets (NiCo-UMOFN) with high OER activity (onset potential of 1.39 V and overpotential of ~189 mV at 10 mA·cm^−2^ under alkaline conditions). In the work of Subramanian et al. [66], nickel and cobalt-based MOFs were constructed using different organic linkers: benzene-1,4-dicarboxylic acid (BDC), 1,3,5-benzene tricarboxylic acid (BTC), and 2-aminobenzene-1,4-dicarboxylic acid (ABDC) as ligands. NiCo-ABDC exhibited the best performance in the OER reaction among the tested systems, with a small onset overpotential (330 mV) and a lower Tafel slope (88 mV·dec^−1^) compared with other metal oxides and MOFs. Bimetallic MIL-53(Co-Ni)/NF for the oxygen evolution reaction, presented by Hu et al. [67], showed excellent OER performance with an ultralow overpotential of 197 mV at a current density of 20 mA·cm^−2^.

**Figure 6:**
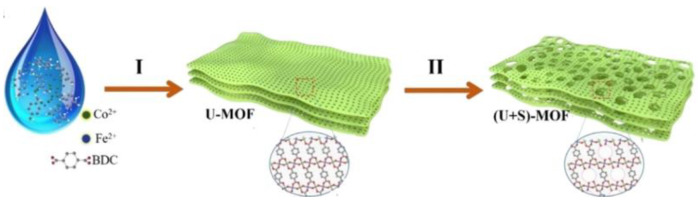
Schematic illustration of the preparation steps and structural evolution of hierarchical 2D CoFe-MOFs based on ultrasound-assisted synthesis and following solvothermal treatment. I—Ultrasonic. II—Solvothermal. Reprinted from [68] with permission from ACS.

Xu et al. [68] synthesized hierarchical 2D CoFe-MOFs in two steps as an OER catalyst (a diagram of the synthesis and evolution of the material is shown in Figure 6). In this system, an increase in electrocatalytic activity for the OER was observed alongside a growth in crystallinity. The catalyst exhibited a low overpotential of 277 mV at 10 mA·cm^−2^ and a Tafel slope of 31 mV·dec^−1^.

In addition, Ling et al. [69] studied bimetallic MOFs containing iron and cobalt and synthesized them as Fe_2_Co MOF/NF using a one-pot hydrothermal method. The catalyst exhibited considerable OER activity with a lower overpotential (224 mV at 10 mA·cm^−2^) and a lower Tafel slope (45.3 mV·dec^−1^) in alkaline media compared with single-metal catalysts (Fe-MOF/NF and Co-MOF/NF). The group of Professor Lin presented research on the use of bimetallic NiFe MOFs in the OER [70]. They synthesized MIL-53 (FeNi)/NF, exhibiting excellent OER activity with a high current density of 50 mA·cm^−2^ at an overpotential of 233 mV, a Tafel slope of 31.3 mV·dec^−1^, and excellent stability in an alkaline aqueous solution. Yet another MOF with these metals was studied for its OER application by Zheng et al. [71]. These studies focused on Ni-BTC MOFs (BTC: 1,3,5-benzenetricarboxylic acid) doped with various Fe contents and the best performance exhibited FeNi_3_-BTC (overpotential of 236 mV at 10 mA·cm^−2^ and a small Tafel slope of 49 mV·dec^−1^). The OER was also investigated using NH_2_-MIL-88B(Fe_2_Ni) MOF on NF [72], which showed extraordinary electrocatalytic activity with an ultralow overpotential of 240 mV at a current density of 10 mA·cm^−2^. This material also offered great mechanical and electrocatalytic stability at a practically favorable high current density of 500 mA·cm^−2^. It has also been shown that the structure of bimetallic MOFs is often reconstructed during the OER reaction into compounds that are more active in this reaction. Zhao et al. [73] revealed that Ni_0.5_Co_0.5_-MOF-74 undergoes a dynamic two-phase structural transformation during the oxygen evolution reaction (OER), forming highly active Ni_0.5_Co_0.5_OOH_0.75_ with oxygen vacancies under high potentials, which significantly enhances catalytic performance. The catalytic material obtained through reconstruction achieved a low overpotential of 198 mV at 10 mA·cm^−2^. A comparison of the electrocatalytic properties of bimetallic MOFs is presented in Table 4.

### 4.3. Trimetallic MOFs and High-Entropy MOFs

**Figure 7:**
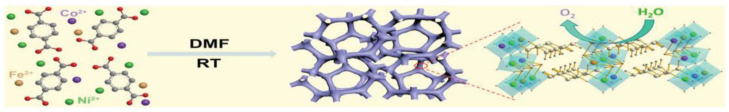
The scheme of formation of hierarchical (Ni_2_Co)_1−x_Fex-MOF-NF at ambient temperature. Reprinted from [74] with permission from Wiley.

Another type of material used as a catalyst in the OER reaction is that of trimetallic MOFs. In 2019, Qian et al. [74] presented research on the use of a hierarchical MOF made from 1,4-benzenedicarboxylate acid (H_2_BDC) as an organic ligand, along with a transition metal (Ni, Co, and Fe). The synthesis scheme is presented in Figure 7. The obtained (Ni_2_Co)_0.925_Fe_0.075_-MOF exhibited the best electrocatalytic properties among others, reaching the lowest overpotential of 257 mV at a current density of 10 mA·cm^−2^, with negligible activity attenuation observed after long-term testing. Furthermore, Wang et al. [75] demonstrated the use of a trimetallic MOF catalyst in this reaction. They prepared NiCo/Fe_3_O_4_/MOF-74, which delivered a remarkably stable OER current with an overpotential as low as 238 mV at 10.0 mA·cm^−2^ and a Tafel slope of 29 mV·dec^−1^.

The use of trimetallic MOFs in OERs made of other metals has also been investigated. Ma et al. [76] prepared CoNi-Cu(BDC) (BDC—1,4-benzenedicarboxylate) and demonstrated good OER performance, with a low overpotential of 327 mV at a current density of 10 mA·cm^−2^ and a small Tafel slope of 75.7 mV·dec^−1^. The trimetallic Co-Ni-Mn metal–organic framework, investigated by Taherinia et al. [77], was synthesized using 1,3,5-tricarboxylic acid as the linker. CoNiMn-MOF exhibited the highest electrocatalytic activity for OER, with an overpotential of 220 mV at a current density of 20 mA·cm^−2^, a Tafel slope of 66 mV·dec^−1^, and excellent electrochemical stability for 20 h.

The 2D and 1D structures of trimetallic MOFs have also been studied. Two-dimensional ultrathin MOF nanosheets made of Ni, Co, and Fe, as well as benzene dicarboxylate (BDC) ligands, demonstrated excellent electrocatalytic OER activity under alkaline conditions, with a low overpotential of 245 mV at 10 mA·cm^−2^. Additionally, 1D CoNiFe-based ZIF microfibers achieved a low overpotential of 273 mV at a fixed current density of 10 mA·cm^−2^. There are also studies on the use of MOFs with a wider variety of metal centers as catalysts for the OER reaction. An example of such materials is the high-entropy metal–organic framework (HE-MOF) [78]. Xu et al. investigated a material composed of five metal elements (Ni, Co, Fe, Zn, and Mo) and a 2,6-naphthalenedicarboxylate tetrahydrate ligand, synthesized using a mild solvothermal method. This HE-MOF, composed of five metals, can cause a synergistic effect, resulting in excellent electrocatalytic OER activity, with an overpotential of 254 mV at a current density of 50 mA·cm^−2^. A comparison of the electrocatalytic properties for OER of catalysts based on tri- and more metallic MOFs is presented in Table 5.

### 4.4. MOFs Modified with Metal Nanoparticles

**Figure 8:**
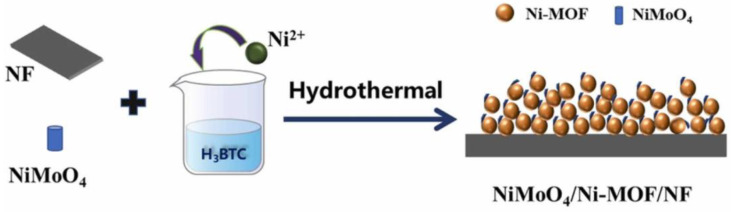
Schematic illustration of the synthetic route for NiMoO4/Ni-MOF/NF as OER catalyst. Reprinted from [81] with permission from Science Direct.

Another way to modify MOF structures used as catalysts in the OER is to decorate them with nanoparticles (NPs). The use of transition metal NPs to decorate zeolitic imidazolate frameworks was explored by Shen et al. They presented a synthesis method for self-supporting ZIF-67/NiCo-S nanoparticle arrays grown directly on nickel foam (NF) frameworks for OER. The OER performance of ZIF-67/NiCo-S/NF was better than that of NiCo-S/NF and ZIF-67-S/NF, demonstrating that the synergistic effect played an important role in the catalytic OER process (requiring a 127 mV overpotential to achieve a current density of 10 mA·cm^−2^ at 1.0 M KOH). Li et al. [81] investigated Ni-MOF grown on nickel foam (NF) by a facile solvothermal method, using 1,3,5-benzenetricarboxylic acid (H_3_BTC) as an organic ligand. NiMoO_4_ nanorods were introduced as dopants onto the homogeneous spherical Ni-MOF (Figure 8) to improve electrical conductivity by stimulating the generation of numerous defects. The resulting composite catalyst, NiMoO_4_/Ni-MOF, exhibited remarkable OER performance in 1.0 M KOH with an overpotential of 218 mV to achieve a current density of 10 mA·cm^−2^ and a Tafel efficiency of 67.75 mV·dec^−1^.

The copper MOF structures decorated with copper oxide were investigated by Amiri et al. [82]. MIL-53(Cu) was synthesized via the hydrothermal method using 1,4-benzenedicarboxylic acid as the organic precursor, followed by thermal treatment to grow CuO hair-like nanostructures on the MOF. The catalyst demonstrated good performance in OER, with an overpotential of 336 mV and a Tafel slope of 64 mV·dec^−1^.

**Figure 9:**
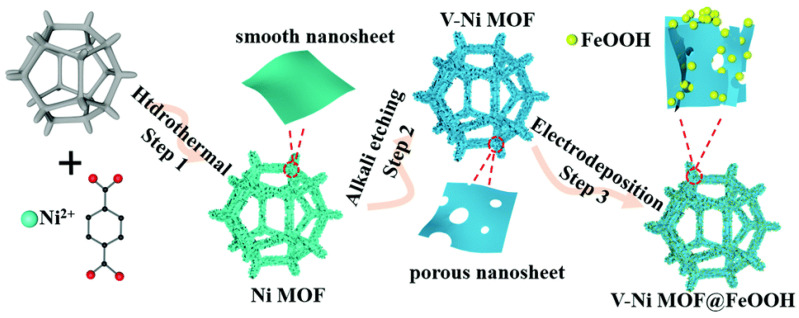
Schematic illustration of the synthetic process of V-Ni-MOF@FeOOH compounds. Reprinted from [84] with permission from RSC.

MOFs for the OER process are also decorated with noble metals. An example of such an application is the work of Cheng et al. [83], who obtained a trimetallic MOF (Fe, Co, Ni) decorated with silver nanoparticles (3 nm in size). Through optimal Au nanoparticle decoration, a significant enhancement in OER performance was achieved on the (FCN)MOF/NP electrode, with the overpotential decreasing from 242 to 216 mV at 10 mA·cm^−2^ and the Tafel slope reducing from 42.1 to 31.7 mV·dec^−1^ [83]. In OER studies, efforts have been made to use MOFs decorated with amorphous nanoparticles. A defect-rich porous Ni MOF, decorated with amorphous FeOOH nanoparticles, was studied by Junan et al. [84] (Figure 9). This composite exhibited superior OER performance in alkaline media, requiring only a small overpotential of 267 mV to reach a current density of 20 mA·cm^−2^. It also exhibited a small Tafel slope of 79 mV·dec^−1^ and remarkable stability. There was a synergistic effect between the defect-rich porous Ni MOF and the amorphous FeOOH nanoparticles. The same research group also investigated bimetallic MOFs (Ni, Fe) decorated with FeOOH nanospheres [85]. The catalyst they developed for OER showed a strong synergistic effect, with excellent OER performance at a small overpotential of 310 mV at 40 mA·cm^−2^.

Wen et al. [86] synthesized a 2D nanosheet material (Fe@BIF-73-NS) that exhibited excellent electrocatalytic performance for the OER reaction of oxygen evolution (overpotential of 291 mV at current density of 10 mA·cm^−2^). Table 6 shows the comparison of electrocatalytic properties for MOF structures together with nanoparticles.

### 4.5. MOF Composites with Graphene and Other Carbon Materials

**Figure 10:**
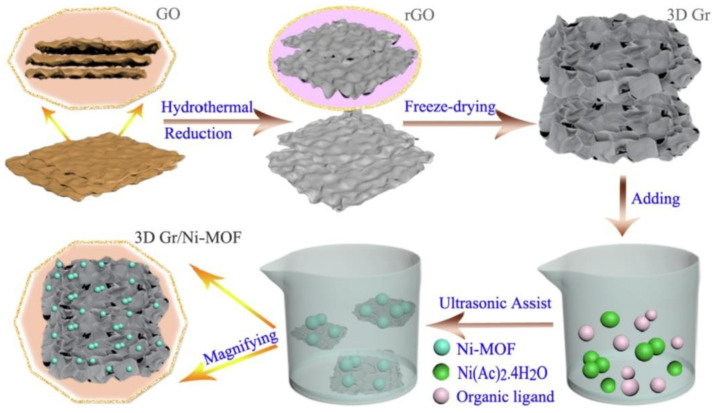
Schematic illustration of the fabrication process of MOF composite with graphene. Reprinted from [89] with permission from Science Direct.

One strategy for modifying MOF structures to increase their conductivity, and consequently their performance as electrocatalysts in the OER, is to dope them with graphene or its derivatives [58]. In 2013, Professor Loh’s group presented research on a graphene oxide and copper-centered metal–organic framework composite (GO-CuMOF) as a trifunctional catalyst for HER, OER, and ORR [88]. The composites exhibited smaller overpotentials and higher currents for all three electrocatalytic reactions and showed better stability in acidic media compared with pure MOF. MOF composites with 3D graphene for OER were developed by Luo et al. [89,90]. They studied MOFs with various transition metals, including both monometallic (a nickel-based MOF, with a diagram of the fabrication of this catalyst shown in Figure 10) and bimetallic systems (cobalt- and copper-based bimetallic MOFs). For systems doped with 3D graphene, better electrocatalytic efficiency was obtained compared with the undoped ones. Additionally, synergistic effects between graphene and the MOF were demonstrated.

Hai et al. [91] presented DFT calculations and synthesis for ultrathin MOF nanosheets (nickel-based with terephthalic acid as the ligand) on graphene. The strategy they proposed allowed for the rapid identification and development of new hybrid catalytic materials for OER reactions. The bimetallic porphyrinic MOF with reduced graphene oxide nanosheets as a highly efficient 2D electrocatalyst for the OER was studied by Meng et al. [92]. The optimized Co–CuTCPP/rGO-10 showed high electrocatalytic activity with a low overpotential of 396 mV at 10 mA·cm^−2^ in an alkaline solution.

Bimetallic Fe_2_Ni MIL-88 with reduced graphene oxide nanocomposite as a catalyst for OER was studied by Abazari et al. [93]. This composite worked effectively in the OER, resulting in a low overpotential of 264 mV at a current density of 10 mA·cm^−2^ and with a Tafel slope of 62 mV·dec^−1^. These studies also showed a positive effect of the MOF structure on reducing graphene oxide agglomeration, which enhanced the accessibility of catalyst surfaces for the aqueous electrolyte, resulting in superior OER electrocatalytic activity in alkaline media.

Chae et al. [94] synthesized a Fe-doped MOF-assisted (Prussian blue analogue) CuCoSe nanostructure on hollow carbon nanofibers (HCNFs) with multifunctional electrocatalytic activity towards OER, HER, and ORR for water splitting applications [94]. Such a catalyst showed good electroactivity in the OER (overpotential of 260 mV at a current density of 20 mA·cm^−2^).

In 2021, Yaqoob et al. [95] prepared, via single-step solvothermal method, a bimetallic iron-nickel 2-amino-terephthalic acid metal–organic framework (FeNiNH_2_BDC MOF) with different weight percentages of carbon nanotubes (CNTs). The best performance exhibited catalysts containing 5 wt.% of CNTs (Tafel slope was 68.50 mV·dec^−1^). The summary of the electrochemical performance of MOFs composites with nanocarbons is presented in Table 7.

### 4.6. MOF-Derived Hybrid Materials

**Figure 11:**
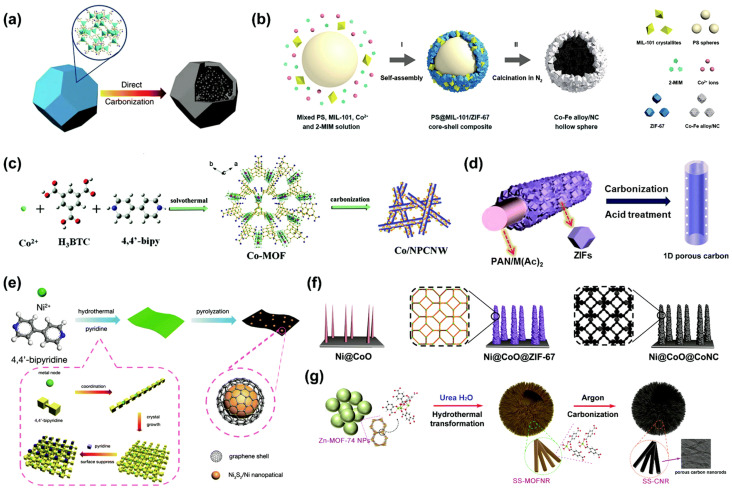
Schematic illustrations of MOF-derived carbon matrices with morphology inherited from MOF precursors. (**a**) Zero-dimensional N-doped microporous carbon polyhedra. (**b**) Zero-dimensional hollow carbon spheres. (**c**) One-dimensional nitrogen-doped porous carbon nanowires. (**d**) One-dimensional nitrogen-doped porous carbon hollow tubules. (**e**) Two-dimensional nitrogen-doped carbon nanoplates. (**f**) Three-dimensional hierarchical nitrogen-doped porous carbon arrays. (**g**) Three-dimensional spherical superstructure of carbon nanorods. Reprinted from [98] with permission from RCS.

MOFs are considered ideal precursors and templates for the formation of advanced electrocatalysts for OER [96]. Their carbon derivatives, obtained through pyrolysis, show high electron conductivity; therefore, they may be used in the OER [97]. During the carbonization process, the organic part transforms into a porous graphitic carbon matrix, and the metal can be converted into metal nanoparticles, metal compound nanoparticles, or atomic metal doping in carbon materials [98]. Many porous carbon materials with controlled morphologies, ranging from 0D to 3D, have been successfully derived from the carbonization of MOFs, highlighting their versatility as precursors. In addition, MOF-derived carbons can be doped with elements such as nitrogen, phosphorus, or sulfur to improve their functional performance [99]. Zhou et al. [64] compared the electrocatalytic activity of catalysts built from transition metals with phosphides, oxides, and sulfides. Their results show that the catalytic performance followed the following order: phosphide > sulfide > oxide. Due to the geometry of the structure, chemical compounds that are derivatives of MOFs are divided into low-dimensional nanostructures, hollow nanostructures, core–shell nanostructures, and hierarchical nanostructures [98]. Figure 11 presents the types of MOF-derived electrocatalysts used in OER.

The use of the zirconium MOF structure (UiO-66) to produce small-sized nanostructures for bifunctional catalysts (OER/ORR) has been discussed by Ahsan et al. [100]_._ From this metal–organic framework, the following structures were created: zero-dimensional (0D) ZrO_2_ (C/ZrO_2_) nanoparticles (NP), BC/ZrO_2_, and heteroatom-doped two-dimensional (2D) carbon nanostructures CN/ZrO_2_. The obtained structures showed high activity as electrocatalysts in the OER (where BC/ZrO_2_ and CN/ZrO_2_ nanohybrids exhibited overpotentials and Tafel slope values of 449 mV and 170 mV·dec^−1^, 409 mV and 140 mV·dec^−1^, and 372 mV and 120 mV·dec^−1^, respectively, at a current density of 10 mA·cm^−2^).

In 2015, Li et al. [101] demonstrated dual high-performance catalysts for OER and ORR through the carbonization and oxidation of MOFs supported on mesoporous graphitic carbon layers. The unique hybrid material derived from MOF-MWCNTs endowed Co_3_O_4_@C-MWCNTs with catalytic performance much better than that of traditional OER materials. An overpotential of 320 mV was required to achieve a current density of 10 mA·cm^−2^.

Li et al. [102] also created two-dimensional Ni-based electrocatalysts (FeNi@CNF and CoNi@CNF) derived from nanosheet-assembled, nanoflower-like MOFs for OER. The nanocarbon porous catalyst formed from the MOF retained the morphology of the precursor material (MOF). Of the two catalysts created, FeNi@CNF exhibited a better overpotential of 356 mV at 10 mA·cm^−2^ with a Tafel slope of 62.6 mV·dec^−1^. Furthermore, FeNi@CNF showed excellent stability for continuous operation at 10 mA·cm^−2^ for 24 h.

Cobalt-containing nanofiber carbon catalysts derived from the ZIF-8/ZIF-67 compound for OER were studied by Wenming Zhang et al. [103]. They compared the pyrolysis temperatures from 700 to 1000 °C, with the best results obtained at the latter. The OER performance of CoNC-CNF-1000 outperformed commercial Pt/C and most nonprecious metal catalysts developed so far. In addition, ZIF-67 was used by Feng et al. as a precursor to create a hybrid CoP/NC catalyst for bifunctional oxygen electrocatalysis. The hybrid was annealed at different temperatures (600, 700, 800, and 900 °C), and phosphidation was performed to obtain the structure of ultrasmall ZIF-67-derived CoP in situ incorporated into nitrogen-doped carbon nanofibers (CoP/NC). The electrocatalytic performance of OER for a catalyst annealed at 800 °C was much higher than that of analogous catalysts derived from ZIF-67, with an overpotential of 290 mV and a low Tafel slope of 62 mV·dec^−1^. Research on the use of Ni-Co_2_O_4_ spinel (synthesized from ZIF-67) as a catalyst in OER, led by Tanget al. [104], showed that the high activity and stability of this catalyst were due to its large active surface area and highly porous structure. The porous NiCo_2_O_4_ demonstrated good activity and stability in OER, with great potential for replacing noble metal catalysts.

MOFs decorated with rare earth metals (La, Ce, and Nd) were also used as precursor catalysts for the OER reaction. Zhang et al. [105] showed the influence of La, Ce, and Nd on the apparent activity and specific activity of the ZIF-67 derivative Co/NC electrode. Their research revealed that lanthanum and neodymium are preferable compounds for the Co active centers at the surface, while cerium donates electrons to the host Co/NC structure [105]. Furthermore, Zhou et al. [106] studied the derivatives of MOFs with lanthanum as a potential catalyst for OER. They synthesized and characterized the composite, La_2_O_3_-Co/AB catalyst (AB = acetylene black), that acts as a bifunctional catalyst for ORR and OER.

The high surface area of hollow nanostructures offers many available active sites, and, when compared with other nanostructured electrocatalysts with high surface area, shows strong confinement effects. Hollow-structured electrocatalysts with porous shells present an important advance in protecting particles from migration and aggregation. Catalysts based on such materials can have three types of architecture: single-shelled, multi-shelled, and open features. The active phases can either be loaded on hollow hosts or integrated into hollow nanoreactors [107].

Ding et al. [108] studied the use of imidazole and CO-based MOF structures (ZIF-67) as precursors to hollow nitrogen-doped carbon polyhedra, including core–shell Co/Co_3_O_4_, yolk–shell Co@Co_3_O_4_, and hollow Co_3_O_4_ nanoparticles. The optimal Co_3_O_4_/HNCP-40 (hollow nitrogen-doped carbon polyhedra formed after 40 min of oxidation) showed significantly high OER activity, with a small overpotential of 333 mV to reach a current density of 10 mA·cm^−2^ [108]. Cobalt MOFs were also used as precursors for catalyst synthesis by Liu et al. [109]. They fabricated CoSe_2_ microspheres with hollow interiors. The optimized CoSe_2_-450 (heated in an argon atmosphere from room temperature to 450 °C) microspheres exhibited excellent OER electrocatalytic activity (overpotential of 330 mV for 10 mA·cm^−2^) with a small Tafel slope of 79 mV·dec^−1^ [109].

Another hollow structure derived from MOF and investigated as a potential OER catalyst is layered double hydroxides (LDHs). Shi et al. [110] studied hollow architecture arrays constructed from NiCo-LDH nanosheets on nickel foam (NiCo-LDH/NF). They used ZnCo-MOF nanopolyhedra as a precursor. The NiCo-LDH/NF sample showed good OER performance in alkaline media, generating high current densities of 100 and 400 mA·cm^−2^, very low overpotentials of 303 and 389 mV, and high stability at 50 mA·cm^−2^ [110]. Additionally, bimetallic cobalt-zinc MOFs were used as precursors for catalysts in OER, as investigated by Chen et al. [111]. They developed an MOF-derived hybrid nanostructure consisting of multiple transition-metal phosphide (NiCoZnP) nanoclusters and hierarchical ultrathin N-doped carbon (NC) nanosheets, which acted as binder-free bifunctional electrocatalysts for overall water splitting. The catalyst exhibited good OER activity (overpotential of 228 mV to reach a current density of 10 mA·cm^−2^).

Research on the use of MOFs containing iron ions as hollow nanostructure promoters for OER catalysts was presented by Wang et al. [112]. They developed a method for synthesizing hollow FeOOH polyhedra using MIL-53(Fe) particles as templates. This material demonstrated the following electrocatalytic properties in the OER reaction: overpotential of 310 mV at 10 mA·cm^−2^ and a Tafel slope of 70 mV·dec^−1^, clearly outperforming FeOOH and Ni(OH)_2_. A comparison of the electrocatalytic properties for the OER reaction of catalysts based on MOF derivatives is presented in Table 8.

## 5. Conclusions

In conclusion, this work reviewed modern materials with potential use as catalysts in the oxygen evolution reaction. Currently, catalysts based on noble metals are still the best performers in the OER, owing to their high stability and activity in acidic environments. However, their high cost and limited availability restrict their widespread application. Therefore, it is important to explore alternative materials that could reduce the cost of this reaction. A much cheaper alternative to noble metal-based catalysts is the use of transition-metal catalysts, which can be employed as metal oxides or composites. However, these catalysts are limited to use in alkaline environments, as their activity declines rapidly in acidic and neutral conditions due to the high potential required.

Another promising class of materials for commercial application in the OER are metal–organic frameworks (MOFs) and their derivatives. The primary advantages of these materials include their high surface area and high concentration of metal centers. However, the main drawback of pure MOF structures in the OER is their poor electrical conductivity. Consequently, research is being conducted to enhance this feature of MOFs. One method is the use of bimetallic, trimetallic, or high-entropy structures. Another approach to improving conductivity is to incorporate MOF structures with nanoparticles, graphene, or other carbon derivatives. Due to their unique structure, MOFs can also serve as precursors to create highly effective catalysts for the OER. These MOF-derived carbon catalysts retain the specific structure of the original MOF materials but exhibit much higher conductivity.

OER electrocatalysis is a crucial component and a stepping-stone for a wide range of electrochemical technologies. The development of low-cost OER catalysts with industrially relevant activity and long-term durability is highly desirable, but this remains a challenge at the current stage. An effective future water-splitting device using MOF-based catalysts must meet the following essential criteria:

1. Low overpotential (<200 mV at 10 mA·cm^−2^)—Enhanced MOFs, such as NiFe-MOFs, must demonstrate strong catalytic performance for both the oxygen evolution reaction (OER) and the hydrogen evolution reaction (HER) to minimize energy consumption.
2. Long-term stability (>1000 h operation)—The MOF structure must maintain its integrity through electrochemical reconstruction, preventing degradation or dissolution within the electrolyte.
3. High faradaic efficiency (~100%)—The device should efficiently convert electrical energy into gaseous H_2_ and O_2_ without side reactions.
4. Compatibility with renewable energy sources—The system should operate under variable voltage (e.g., from photovoltaics) while optimizing energy consumption.
5. Scalable electrode production—The process for manufacturing MOF-coated conductive substrates (e.g., carbon or metal-based) must be cost-effective for industrial applications.

A major challenge remains the integration of MOFs with membrane systems (e.g., proton exchange membrane (PEM)) to enable efficient gas separation and prevent crossover.

## Figures and Tables

**Table 1:** Comparison of the electrocatalytic efficiency in OER over different noble metal-based catalysts.

Catalyst	Electrolyte	Overpotential (mV) at (10 mA·cm^−2^)	Tafel Slop (mV·dec^−1^)	Reference
Ru-branched nanoparticles	0.1 M HClO_4_	180	52	[31]
Ru nanoparticles	0.5 M H_2_SO_4_	202	70	[32]
Ru-NiWNx	1 M KOH	270	79	[33]
RuO_2_ nanosheets	0.5 M H_2_SO_4_	199	38	[34]
Re_0.06_Ru_0.94_O_2_	0.1M HClO_4_	190	45	[30]
Mn–RuO_2_	0.5 M H_2_SO_4_	158	43	[24]
3R-IrO_2_	0.1 M HClO_4_	188	52	[23]
Ir@N-G-750	0.5 M H_2_SO_4_	303	50	[27]
Ir aNCs@C	0.5 M H_2_SO_4_	290	55	[28]
Ir NTs	0.1 M HClO_4_	245	49	[35]
IrCo hollow nanospheres	0.5 M H_2_SO_4_	284	56	[36]
RuIrTe NTs	0.5 M H_2_SO_4_	205	41	[37]
IrO_x_-TiO_2_-Ti	0.5 M H_2_SO_4_	200	49	[38]
Co-RuIr	0.1 M HClO_4_	235	31	[39]
M-RuIrFeCoNiO_2_	0.5 M H_2_SO_4_	262	49	[29]

**Table 2:** Comparison of electrocatalytic efficiency of transition metal-based catalysts in OER.

Catalyst	Electrolyte	Overpotential (mV) at (10 mA·cm^−2^)	Tafel Slope (mV·dec^−1^)	Reference
Co_3_O_4__SBA15	1 M KOH	440	65	[40]
Co_3_O_4_/BOx nanowire	1 M KOH	328	86	[41]
Zn_0.6_Co_0.4_Al_2_O_4_	0.1 M KOH	440	72	[55]
Te-Co_3_O_4_	1 M KOH	313	75	[42]
Co-Fe-B-P	1 M KOH	294	49.5	[56]
MnFe_2_O_4_/NF	1 M KOH	310	65	[46]
Co_3_O_4_ spheres	1 M KOH	308	82	[48]
Co_3_O_4_ spheres 125 Mt	1 M KOH	252	26.7	[48]
Co_3_O_4_/mesoporous carbon	0.1 M KOH	360	91	[44]
CoS nanosheets	1 M KOH	310	55	[49]
CuCo_2_S_4_nanosheets	1 M KOH	310	86	[50]
Ni_2_P	1 M KOH	250	60	[52]
Fe–Co–P/NF	1 M KOH	227	55	[54]

**Table 3:** Comparison of electrocatalytic efficiency of monometallic MOF catalysts in OER.

Catalyst	Electrolyte	Overpotential (mV) at (10 mA·cm^−2^)	Tafel Slope (mV·dec^−1^)	Reference
UTSA-16	1.0 M KOH	408	77	[59]
Co-MOF-74	0.1 M KOH	337	-	[60]
ZIF-67 200	1 M KOH	318	105	[62]
BIF-90	0.1 M KOH	460	-	[63]
Fe-MOF/NF	1 M KOH	240	72	[61]

**Table 4:** Comparison of electrocatalytic efficiency of bimetallic MOF catalysts in OER.

Catalyst	Electrolyte	Overpotential (mV) at (10 mA·cm^−2^)	Tafel Slope (mV·dec^−1^)	Reference
NiCo-BDC	1 M KOH	370	98	[66]
NiCo-BTC	1 M KOH	370	92	[66]
NiCo-ABDC	1 M KOH	330	88	[66]
CTGU-10c2	0.1 M KOH	240	58	[64]
NiCo-UMOFNs	1 M KOH	189	42	[65]
MIL-53(Co-Ni)	1 M KOH	197	85	[67]
hierarchical 2D CoFe-MOF	1 M KOH	277	31	[68]
Fe_2_Co MOF/NF	1 M KOH	224	45	[69]
MIL-53(FeNi)/NF	1 M KOH	233	31	[70]
FeNi_3_-BTC	1 M KOH	236	49	[71]
FeNi_10_-BTC	1 M KOH	277	60	[71]
FeNi-BTC	1 M KOH	257	50	[71]
NFN-MOF/NF	1 M KOH	240	59	[72]
Ni_0.5_Co_0.5_OOH_0.75_	1 M KOH	198	49	[73]

**Table 5:** Comparison of the electrocatalytic efficiency of trimetallic MOFs and HE-MOFs in OER.

Catalyst	Electrolyte	Overpotential (mV) at (10 mA·cm^−2^)	Tafel Slope (mV·dec^−1^)	Reference
(Ni_2_Co_1_)_0.925_Fe_0.075_-MOF	1 M KOH	257	41	[74]
NiCo/Fe_3_O_4_/MOF-74	1 M KOH	238	29	[75]
CoNi-Cu(BDC)	1 M KOH	327	75	[76]
CoNiMn-MOF	1 M KOH	220	66	[77]
2D-(Ni_3_Co_1_)_3_Fe_1_-MOFNSs	1 M KOH	245	51	[79]
1D-CoNiFe-ZIF-MF	1 M KOH	273	87	[80]
HE-MOF	1 M KOH	254	61	[78]

**Table 6:** Comparison of the electrocatalytic efficiency MOF decorated with NPs in OER.

Catalyst	Electrolyte	Overpotential (mV) at (10 mA·cm^−2^)	Tafel Slope (mV·dec^−1^)	Reference
ZIF-67/NiCo-S	1 M KOH	127	80	[87]
NiMoO_4_/Ni-MOF	1 M KOH	218	68	[81]
CuO@MIL-53(Cu)	1 M KOH	336	64	[82]
Au/(FCN)MOFs	1 M KOH	216	32	[83]
Ni-MOF@FeOOH	1 M KOH	267	79	[84]
Fe-MOF@FeOOH	1 M KOH	303	36	[85]
Fe@BIF-73-NS	1 M KOH	291	38	[86]

**Table 7:** The electrocatalytic efficiency of MOFs composites with nanocarbons in OER.

Catalyst	Electrolyte	Overpotential (mV) at (10 mA·cm^−2^)	Tafel Slope (mV·dec^−1^)	Reference
GO-CuMOF	Acid	-	65	[88]
3D Gr/Ni-MOF	0.1 M KOH	370	91	[89]
Tem3DGS-CoCu-MOF	0.1 M KOH	460	172	[90]
Ni-HMOF@GE-PBA	1 M KOH	143	42	[91]
Co–CuTCPP/rGO	1 M KOH	393	58	[92]
Fe_2_Ni MIL-88/rGO	1 M KOH	264	62	[93]
Fe-doped MOF CuCoSe@HCNFs	1 M KOH	260	57	[94]
FeNiNH_2_BDC MOF/5%CNTs	1 M KOH	220	68.5	[95]

**Table 8:** Comparison of electrocatalytic efficiency of OER in MOF derived materials.

Catalyst	Electrolyte	Overpotential (mV) at (10 mA·cm^−2^)	Tafel Slope (mV·dec^−1^)	Reference
UIO-66 derived C/ZrO_2_	0.1 M KOH	449	170	[100]
BC/ZrO_2_	0.1 M KOH	409	140	
CN/ZrO_2_	0.1 M KOH	372	120	
BCN/ZrO_2_	0.1 M KOH	301	75	
Co_3_O_4_@C-MWCNTs	0.1 M KOH	320	62	[101]
FeNI@CNF	1 M KOH	356	63	[102]
CoNi@CNF	1 M KOH	388	85	
ZIF-8/ZIF-67 der. CoNC-CNF-1000	0.1 M KOH	-	98	[103]
CoP/NC	0.1 M KOH	290	62	[113]
Co_3_O_4_/HNCP-40	1 M KOH	333	69	[108]
La_2_O_3_-Co/AB	1 M KOH	299	92	[106]
CoSe_2_-450	1 M KOH	330	79	[109]
NiCo-LDH/NF	1 M KOH	303	-	[110]
FeOOH polyhedra	1 M KOH	310	70	[112]

## Data Availability

No new data were created or analyzed in this study. Data sharing is not applicable to this article.
